# A case of mixed neuroendocrine carcinoma–acinar adenocarcinoma: Utilization of triplet therapy for prostate cancer

**DOI:** 10.1002/iju5.12778

**Published:** 2024-09-01

**Authors:** Junichi Ikeda, Hisanori Taniguchi, Hiroki Takayama, Yuki Masuo, Takahiro Nakamoto, Katsunori Uchida, Hidefumi Kinoshita

**Affiliations:** ^1^ Department of Urology and Andrology Kansai Medical University Hirakata Osaka Japan; ^2^ Department of Pathology Kansai Medical University Hirakata Osaka Japan

**Keywords:** darolutamide, metastatic hormone‐sensitive prostate cancer, mixed neuroendocrine carcinoma–acinar adenocarcinoma, neuroendocrine prostate cancer, triplet therapy

## Abstract

**Introduction:**

Neuroendocrine prostate cancer is an aggressive histological subtype of prostate cancer with a poor prognosis. Neuroendocrine prostate cancer is traditionally treated with cisplatin‐based chemotherapy, similar to that used in treating small‐cell lung cancer. However, the therapeutic effectiveness of chemotherapy for neuroendocrine prostate cancer has been limited. This case report describes a response to triplet therapy using darolutamide, androgen deprivation therapy, and docetaxel, which was administered in a patient with mixed neuroendocrine prostate cancer.

**Case presentation:**

A 77‐year‐old man was newly diagnosed with mixed neuroendocrine prostate cancer. Serum prostate‐specific antigen, neuron‐specific enolase, and progastrin‐releasing peptide levels were 62.2, 40.6, and 60.6 pg/mL, respectively. Multiple lymph node metastases were identified on a computed tomography scan, and bone scintigraphy revealed multiple bone metastases. The clinical stage was determined to be cT3bN1M1b. Ultimately, tumor size and serum markers decreased with triplet therapy.

**Conclusion:**

We demonstrated the first case in which triplet therapy had been effective in the treatment of neuroendocrine prostate cancer.

**Abbreviations & Acronyms**
ADT: androgen deprivation therapy
ALT: alanine aminotransferase
AR: androgen receptor
ARSI: androgen receptor signaling inhibitor
AST: aspartate aminotransferase
CI: confidence interval
CRPC: castrate‐resistant prostate cancer
CT: computed tomography
mCSPC: metastatic castration‐sensitive prostate cancer
MRI: magnetic resonance imaging
NEPC: neuroendocrine prostate cancer
NSE: neuron‐specific enolase
PC: prostate cancer
ProGRP: progastrin‐releasing peptide
PSA: prostate‐specific antigen
T‐NEPC: treatment‐related neuroendocrine prostate cancer

**Keynote message**

We experienced a favorable oncological outcome to triplet therapy using docetaxel in a patient with mixed NEPC.

## Introduction

Recently, combination therapy of ADT plus docetaxel or ARSI has improved overall survival in patients with mCSPC.^1^ Triplet therapy using darolutamide, ADT, and docetaxel is approved for treatment of patients with mCSPC.^2^ In the 2022 World Health Organization classification of urinary and male genital tumors, de novo NEPCs include pure small‐cell, large‐cell, and mixed tumors.^3^ Combinations of carboplatin or cisplatin with etoposide or taxane have been used for NEPC^4^; however, their therapeutic effects have been limited. This is the first case report of a response to triple therapy using darolutamide to treat mixed NEPC.

## Case presentation

**Fig. 1:**
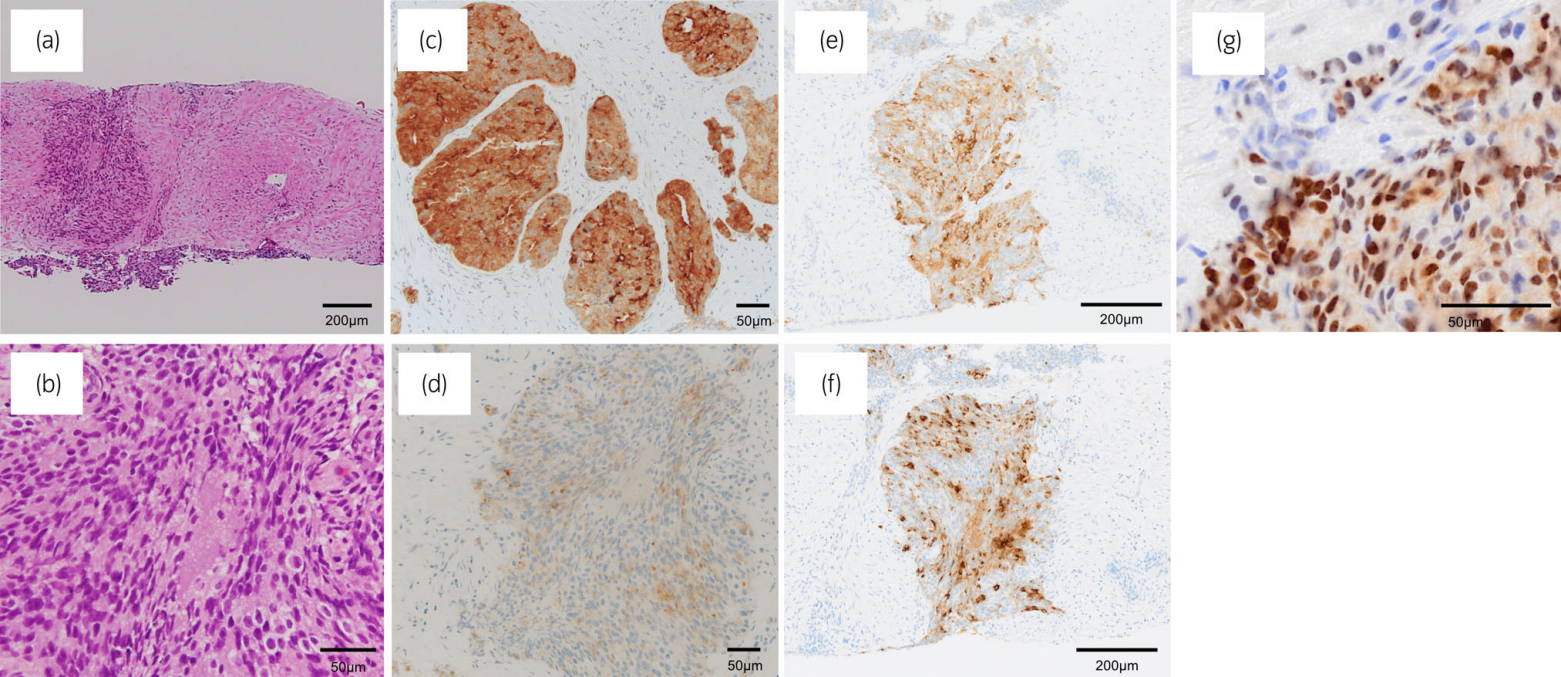
Microscopic images of a needle biopsy specimen. Hematoxylin and eosin staining images of neuroendocrine prostate cancer: low‐power view (a); high‐power view (b); positive PSA staining for acinar adenocarcinoma components (c); negative PSA staining for neuroendocrine carcinoma components (d); neuroendocrine carcinoma components show positive staining for synaptophysin (e), and chromogranin A (f); predominantly positive AR staining for neuroendocrine carcinoma components (g).

**Fig. 2:**
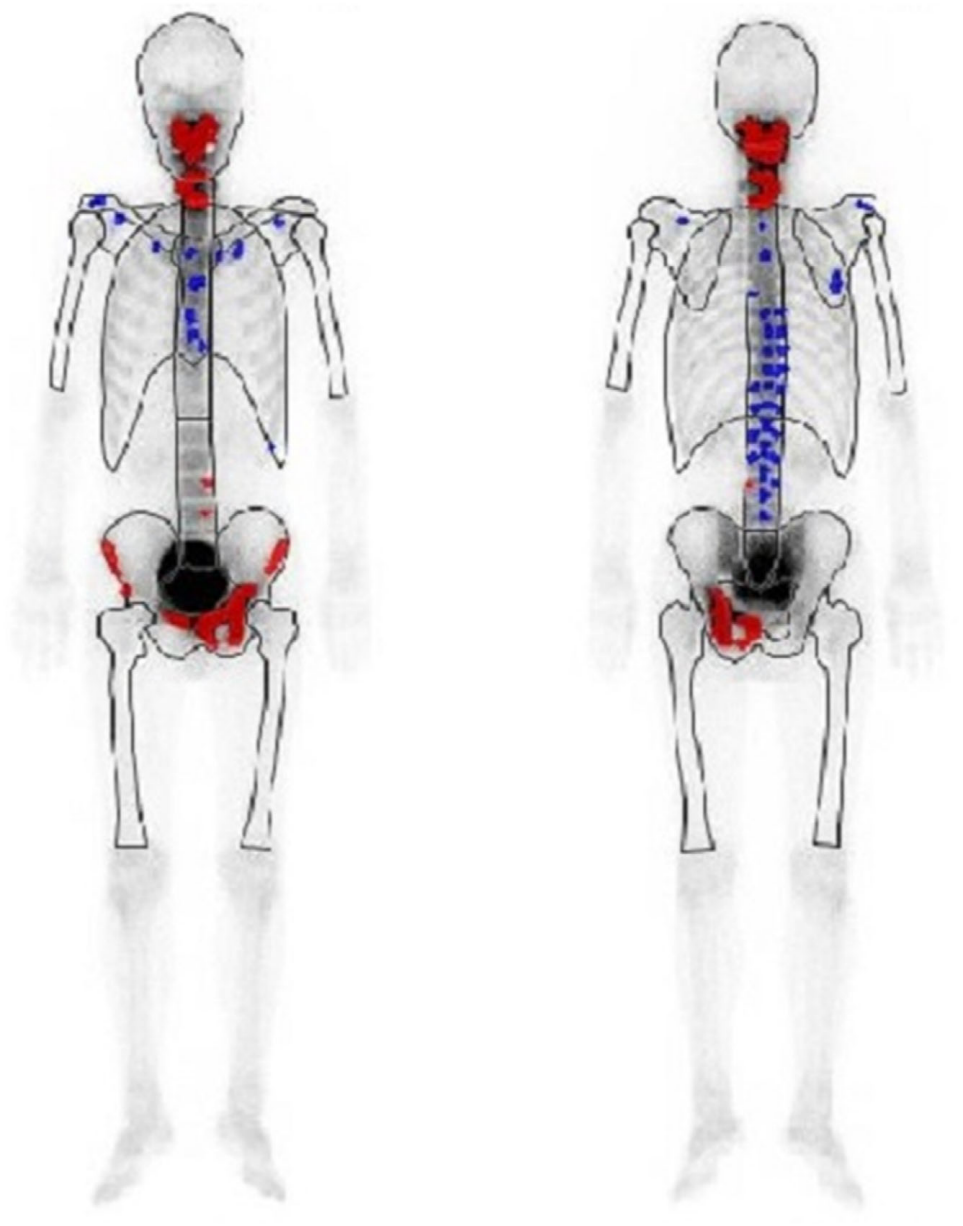
Bone scintigraphy revealed multiple bone metastases in the spine and pelvis.

A 77‐year‐old man presented with neck pain. Multiple bone tumors were detected using MRI scanning. Serum PSA, NSE, and ProGRP levels were 62.2 ng/mL, 40.6 ng/mL (normal level <16.3 ng/mL), and 60.6 pg/mL (normal level <81 pg/mL), respectively. A pelvic MRI demonstrated that the mass had spread to both sides of the prostate, from the peripheral zone to the transition zone, and had also invaded the seminal vesicles and bladder neck. A prostate needle biopsy showed a mixed neuroendocrine carcinoma–acinar adenocarcinoma, with predominantly acinar adenocarcinoma (90% vs 10%) (Fig. 1a,b). Immunohistochemical findings are shown in Figure 1c–g. Multiple lymph node metastases without visceral metastases were identified via CT scanning, and bone scintigraphy revealed multiple bone metastases (Fig. 2).

**Fig. 3:**
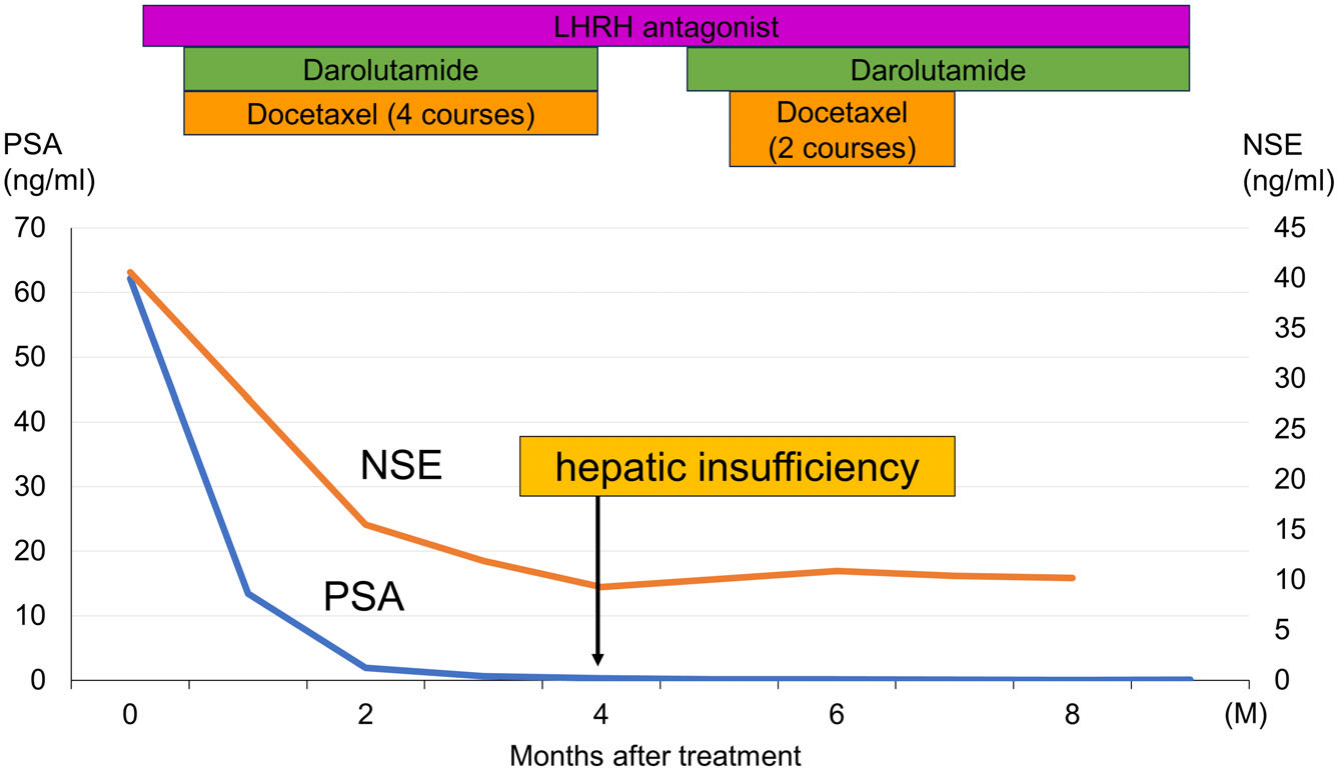
Treatment course and tumor markers. Serum PSA and NSE levels decreased and remained at the nadir. The normal level of NSE is less than or equal to 16.3 ng/mL.

**Fig. 4:**
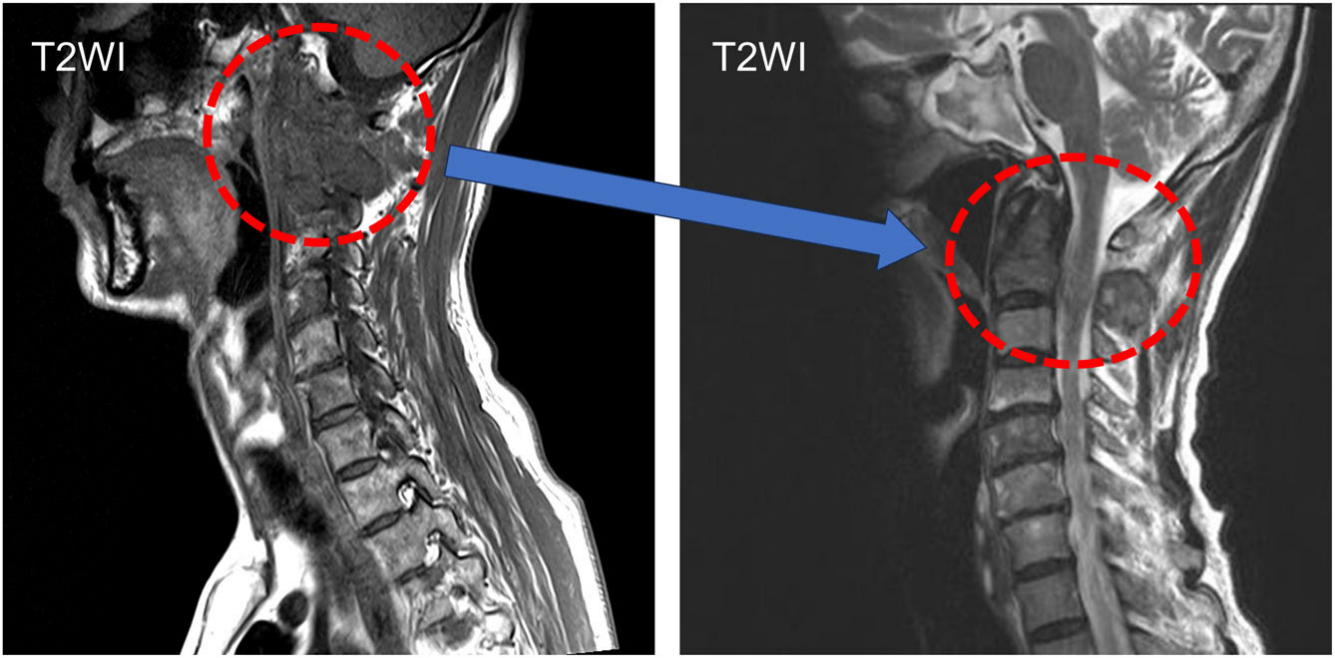
Tumor response to triplet therapy. The tumor in the cervical spine has shrunk significantly.

Triplet therapy using darolutamide, androgen deprivation therapy (degarelix), and docetaxel were administered. Darolutamide was administered at 1200 mg/day. The initial dose of degarelix was 240 mg, with subsequent subcutaneous injections of 480 mg every 3 months. Docetaxel was administered once every 21 days at a dose of 70 mg/m^2^, for a total of 6 courses. Due to neutropenia, granulocyte colony‐stimulating factor was administered for 2–4 days per course of docetaxel. After 4 courses of docetaxel, the darolutamide and docetaxel were withdrawn for 3 weeks due to elevated transaminases (AST/ALT). After this combination was re‐administered, there was no increase in AST and ALT levels. The serum PSA level decreased to 0.081 ng/mL (nadir) within 6 months of starting the treatment, and maintained below measurement sensitivity (Fig. 3). The serum NSE and ProGRP levels also decreased to 10.4and 23.3 pg/mL, respectively. MRI imaging revealed shrinkage of neck metastases (Fig. 4), which coincided with improvement in neck pain. There were no increases in tumor markers after 3 months of ceasing docetaxel treatment.

## Discussion

This study presents a novel case of de novo NEPC that responded well to triplet therapy using darolutamide. NEPC can be subdivided into de novo NEPC and T‐NEPC.^5^ De novo NEPC is rare, accounting for approximately 0.5%–2% of primary PCs. On the other hand, T‐NEPCs occur in about 10%–20% of CRPCs that have been treated with hormone therapy. De novo NEPC has been classified as follows: (1) usual prostate adenocarcinoma with neuroendocrine differentiation; (2) adenocarcinoma with Paneth cell‐like neuroendocrine differentiation; (3) carcinoid tumor; (4) small‐cell neuroendocrine carcinoma; (5) large‐cell neuroendocrine carcinoma; and (6) mixed neuroendocrine carcinoma–acinar adenocarcinoma.^6^ Of these varieties, mixed neuroendocrine carcinoma–adenocarcinomas account for one‐third of all de novo NEPCs.^7^ This study examined a mixed neuroendocrine carcinoma–acinar adenocarcinoma. Androgen deprivation therapy using ARSI was required because the patient was hormone‐naïve, while chemotherapy was also considered necessary due to the small‐cell component.

There is currently no standardized treatment or therapy for NEPC.^5^ In particular, the therapeutic significance and prognostic implications of de novo NEPC are unclear.^8^ To this point, treatment options for NEPC have been similar to those for small‐cell lung cancer.^9^ Specifically, it has been treated using platinum combined with either etoposide and irinotecan, or with docetaxel and amrubicin.^10^ In a small number of cases reported by Abdulfatah *et al*., various treatments have been previously conducted.^8^ These include androgen deprivation therapy, androgen deprivation therapy followed by docetaxel chemotherapy, carboplatin‐based chemotherapy, and radiation with concurrent androgen deprivation therapy.^8^ However, there has been no report of cases using ARSI treatment options, such as triplet therapy.

Recently, the treatment of mCSPC has dramatically changed. The combination of ADT with docetaxel or ARSI has improved overall survival when compared to ADT usage alone.^11^ Furthermore, ARASENS and PEACE‐1 trials in 2022 demonstrated the treatment efficacy of a three‐drug combination therapy that adds a novel AR inhibitor to the ADT and docetaxel.^2^, ^12^ Within the ARASENS trial, triplet therapy using darolutamide showed that the risk of death was 32.5% lower in the darolutamide group than in the placebo group (hazard ratio 0.68; 95% CI, 0.57–0.80; *p* < 0.001).^2^ Overall survival at 4 years was 62.7% (95% CI, 58.7–66.7) in the darolutamide group and 50.4% (95% CI, 46.3–54.6) in the placebo group. In our case, we found triplet therapy to be very effective. It may be the optimal choice of initial therapy for treating de novo NEPC, particularly mixed NEPCs. However, this case report has some limitations. First, it is possible that the serum NSE and pro‐GRP did not reflect the viability of NEPC because the proportion of NEPC was low. The effects of this treatment might better be seen in the limited effects on acinar adenocarcinomas, rather than on NEPCs. Although the neuroendocrine carcinoma components were low, platinum‐based therapy might be preferable if the NEPC component is bulky. Second, this study had a very short follow‐up time and reported only one case. A longer follow‐up, in addition to more data, will be required in future studies.

## Conclusion

To our knowledge, this is the first report of a significant response of NEPC to triplet therapy. This case could serve as a basis for future treatment of mixed NEPC.

## Author contributions

Junichi Ikeda: Conceptualization; writing – original draft. Hidefumi Kinoshita: Supervision. Takahiro Nakamoto: Writing – review and editing.

## Conflict of interest

The authors declare that they have no competing interests.

## Approval of the research protocol by an institutional reviewer board

This study was approved by the relevant ethics committee.

## Informed consent

Not applicable.

## Registry and the Registration No. of the study/trial

Not applicable.
